# Adaptability and Sensitivity of *Trichoderma* spp. Isolates to Environmental Factors and Fungicides

**DOI:** 10.3390/microorganisms13071689

**Published:** 2025-07-18

**Authors:** Allinny Luzia Alves Cavalcante, Andréia Mitsa Paiva Negreiros, Naama Jéssica de Assis Melo, Fernanda Jéssica Queiroz Santos, Carla Sonale Azevêdo Soares Silva, Pedro Sidarque Lima Pinto, Sabir Khan, Inês Maria Mendes Sales, Rui Sales Júnior

**Affiliations:** 1 Department of Agronomic and Forest Sciences, Universidade Federal Rural do Semi-Árido, Mossoró 59625-900, Brazil; andreiamitsa@gmail.com (A.M.P.N.); naama.melo@ufersa.edu.br (N.J.d.A.M.); santosfernanda518@gmail.com (F.J.Q.S.); carla.soares@alunos.ufersa.edu.br (C.S.A.S.S.); pedrosidarque@hotmail.com (P.S.L.P.); 2 Technological Development Center—CDTec, Universidade Federal de Pelotas, Pelotas 96010-610, Brazil; sabir@ufersa.edu.br; 3 Department of Applied Social Sciences, Universidade Federal Rural do Semi-Árido, Mossoró 59625-900, Brazil; inesmariams@gmail.com

**Keywords:** biological control, fitness components, mycelial growth, tolerance

## Abstract

Biological control employs beneficial microorganisms to suppress phytopathogens and mitigate the incidence of associated plant diseases. This study investigated the in vitro development and survival of *Trichoderma* spp. isolates derived from commercial formulations under different temperatures, pH levels, and sodium chloride (NaCl) concentrations and with synthetic fungicides with distinct modes of action. Three isolates were analyzed: URM-5911 and TRA-0048 (*T*. *asperellum*) and TRL-0102 (*T*. *longibrachiatum*). The results revealed substantial variability among the isolates, with the optimal mycelial growth temperatures ranging from 24.56 to 29.42 °C. All the isolates exhibited broad tolerance to the tested pH (5–9) and salinity levels (250–1000 mM), with TRL-0102 demonstrating the highest salt resistance. The fungicide treatments negatively affected mycelial growth across all the isolates, with Azoxystrobin + Difenoconazole and Boscalid causing growth reductions of up to 50%. Notably, Boscalid enhanced conidial production more compared to the control (126.0% for URM-5911, 13.7% for TRA-0048, and 148.5% for TRL-0102) and decreased the percentage of inactive conidia to less than 10% in all the isolates. These results provide strategic information for the application of *Trichoderma* spp. in agricultural systems, supporting the selection of more adapted and suitable isolates for integrated disease management programs.

## 1. Introduction

Biological control represents an ecologically sustainable approach that utilizes beneficial microorganisms to manage and suppress phytopathogens, thereby mitigating the deleterious effects of plant diseases [1]. These microorganisms, commonly referred to as biological control agents (BCAs), can act against a broad spectrum of phytopathogens and play a critical role in promoting plant growth and protecting crops from pathogenic fungi and nematodes [2]. Among the most prominent and widely applied BCAs are fungi of the genus *Trichoderma* [3].

*Trichoderma* is a fungal genus within the phylum Ascomycota characterized by its widespread environmental occurrence and ecological role in the decomposition of plant residues, contributing to nutrient cycling and environmental balance [4]. Approximately 375 *Trichoderma* species have been morphologically and molecularly identified to date [5]. Most species reproduce asexually, producing unicellular conidia and thick-walled chlamydospores that function as survival propagules [6]. Their mechanisms of action are diverse and include antibiosis, competition, mycoparasitism, plant growth promotion, and the induction of systemic resistance against phytopathogens. Additionally, several *Trichoderma* species produce hydrolytic enzymes with potential industrial applications, such as polysaccharidases, proteases, and lipases [7]. These attributes make *Trichoderma* species promising BCAs, known for their environmental safety and non-toxicity to human health [8].

Approximately 50–60% of commercial biofungicides are derived from *Trichoderma* spp., with numerous formulations based on single or multiple isolates currently available worldwide. These products can suppress more than 100 species of fungal phytopathogens [9,10]. Unlike synthetic fungicides, biofungicides are biodegradable, decompose rapidly, and do not accumulate in ecosystems [11,12]. Their application is particularly advantageous in organic farming systems and environmentally sensitive areas where chemical inputs are restricted, providing a sustainable alternative to synthetic fungicides [13,14]. However, their performance can be significantly influenced by environmental conditions and the presence of non-selective chemical fungicides [15].

Soil temperature, pH, and salinity are among the most critical environmental factors affecting the growth and biocontrol efficacy of *Trichoderma* spp. Most microorganisms are mesophilic, with optimal growth and sporulation occurring between 25 and 35 °C. Nevertheless, some species have adapted to extreme environments and can be isolated from both colder and warmer regions worldwide [6,16,17]. Regarding pH, the optimal range for growth and sporulation is between 4.1 and 8.6, with significant reductions outside this interval [17,18]. Acidic conditions generally enhance germination capacity, while alkaline environments tend to inhibit fungal development [19]. Moreover, pH can modulate enzymatic activity and interaction with phytopathogens such as *Sclerotinia sclerotiorum*, *Fusarium solani*, and *Rhizoctonia solani* [20,21,22]. Despite the adverse effects of salinity on fungal growth, many *Trichoderma* species are capable of tolerating sodium chloride concentrations as high as 855 mM of sodium chloride (NaCl) without compromising their saprophytic activity [23,24].

The integration of these BCAs with synthetic fungicides in integrated disease management (IDM) strategies aims to enhance plant disease control, reduce sole dependency on chemical pesticides, and mitigate environmental impacts [25]. However, some synthetic fungicides may inhibit the growth or functionality of BCAs, potentially compromising their biocontrol effectiveness [26,27,28].

These biological and environmental characteristics are essential for selecting *Trichoderma* isolates that are well-adapted to specific cultivation conditions and compatible with the conventional agricultural inputs. Given the dynamic nature of microbial interactions, continuous research is essential to improve our understanding of *Trichoderma* spp. responses to environmental variables and fungicidal compounds (fitness components), with the goal of optimizing their application across diverse agricultural systems. Accordingly, the present study aimed to evaluate the mycelial growth, development, and survival of *Trichoderma* spp. isolates obtained from commercially registered products in Brazil under different temperatures, pH levels, and salinity concentrations and with synthetic fungicides with different modes of action.

## 2. Materials and Methods

### 2.1. Trichoderma *spp.* Isolates

Three *Trichoderma* spp. isolates (URM-5911, TRA-0048, and TRL-0102) obtained from commercial biological products were used in this study (Table 1). These *Trichoderma*-based products were selected based on their use in melon production areas in the northeastern region of Brazil. For isolate recovery, 100 μL aliquots of each *Trichoderma*-based commercial formulation were plated onto Petri dishes containing potato dextrose agar (PDA; Merck KGaA, Darmstadt, Germany) and incubated in a BOD-type growth chamber at 25 ± 1 °C in darkness for 7 to 10 days to allow for fungal development.

### 2.2. Effect of Temperature, pH, and Salinity on the Mycelial Growth Rate of Trichoderma *spp.*

The effects of temperature, pH, and salinity on the mycelial growth rate of the *Trichoderma* spp. isolates were assessed using PDA medium. Mycelial plugs (8 mm in diameter) obtained from the actively growing margins of 7-day-old colonies were transferred to the center of Petri dishes containing PDA (one plug per plate). For the temperature assay, the plates were incubated in the dark at 5, 10, 15, 20, 25, 30, 35, 40, and 45 °C in a controlled-environment growth chamber. For the pH assay, the medium was adjusted to pH 5, 6, 7, 8, and 9 using 1 N sodium hydroxide (NaOH). For the salinity assay, PDA was supplemented with NaCl at concentrations of 0, 250, 500, 750, and 1000 mM [29]. Plates for both the pH and salinity tests were incubated in the dark at 25 ± 1 °C. Colony diameter was measured in two perpendicular directions using a graduated ruler when growth reached at least two-thirds of the plate or by the third day of incubation. These measurements were used to calculate the mycelial growth rate (MGR), expressed in centimeters per day (cm·d^−1^).

### 2.3. Sensitivity of Trichoderma *spp.* to Fungicides

The effects of fungicides on the mycelial growth, conidial production, and isolate viability were evaluated in vitro as described by Khirallah et al. [1]. Five fungicides containing different active ingredients were tested: Azoxystrobin + Difenoconazole (AzD—Amistar Top^®^ CS, systemic, Syngenta Proteção de Cultivos Ltd., Paulínia, Brazil); Metiram + Pyraclostrobin (MeP—Cabrio^®^ Top WG, systemic, BASF S.A., São Paulo, Brazil); Boscalid (Bos—Cantus^®^ WG, systemic, BASF S.A., Brazil); Fludioxonil (Flu—Maxim^®^ CS, contact, Syngenta Proteção de Cultivos Ltd., Brazil); and Difenoconazole (Dif—Score^®^ EC, systemic, Syngenta Proteção de Cultivos Ltd., Brazil).

The fungicides were incorporated into PDA medium at the recommended label rates for each product (AzD = 256 mg·L^−1^; MeP = 4000 mg·L^−1^; Bos = 1600 mg·L^−1^; Flu = 2000 mg·L^−1^; and Dif = 500 mg·L^−1^). After medium solidification, 8 mm diameter mycelial plugs obtained from the actively growing edge of 7-day-old colonies were transferred to the center of Petri dishes containing PDA supplemented with fungicides (one plug per plate). A PDA plate without fungicide was used as a control. All the plates were incubated in a climate-controlled chamber at 25 ± 1 °C in the dark. The diameter of each colony was measured in two perpendicular directions when the colony reached at least two-thirds of the plate or up to the seventh day of growth.

To determine conidial production, four 5 mm diameter mycelial plugs were collected from 7-day-old cultures for each treatment, transferred to Eppendorf-type microtubes containing 1 mL of sterile distilled water, and agitated on an orbital shaker at 30 rpm for 5 min to ensure the complete release of conidia into the suspension. Conidial concentrations for each fungicide treatment were assessed using a Neubauer hemocytometer. To evaluate conidial viability, 20 μL aliquots of the previously obtained conidial suspensions were deposited at five predetermined points on Petri dishes containing PDA. The plates were incubated in a climate-controlled chamber at 25 ± 1 °C in the dark for 10–20 h. After 10 h, a drop of methylene blue was added to each point to halt germination, and the samples were subsequently covered with a coverslip.

**Figure 1:**
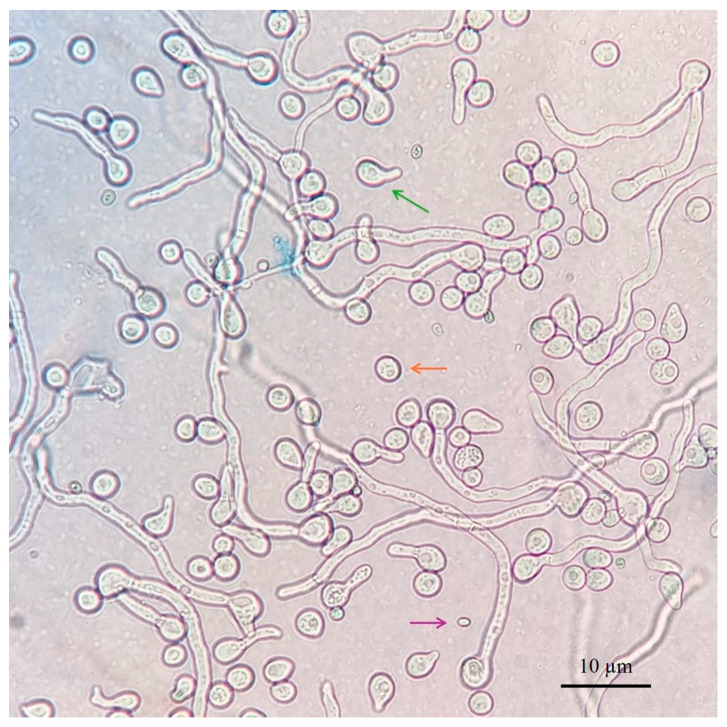
Conidial viability observed under an optical microscope. The germinated active conidia are indicated by the green arrow, the non-germinated active conidia are indicated by the orange arrow, and the inactive conidia are indicated by the purple arrow.

At the end of the incubation period, conidial viability was assessed microscopically by analyzing a sample of 100 conidia per point. Germinated active conidia were defined as those that had initiated the germination process and exhibited a developing germ tube. Non-germinated active conidia were defined as those that had begun the germination process, but did not show a visible germ tube at the time of analysis. Inactive conidia were identified as non-viable, showing no changes in size or morphology (Figure 1).

### 2.4. Experimental Design and Data Analysis

The experimental design followed a completely randomized layout, with 15 replicates for each tested combination of isolate, temperature, pH, salinity, and fungicide. Analysis of variance (ANOVA) was performed for all the datasets. The MGR of each isolate was plotted against temperature and pH using a cubic polynomial regression model (y = a + bx + cx^2^ + dx^3^) and against salinity using a linear regression model (y = a + bx) employing the software TableCurve 2D v. 5.01 (Systat Software Inc., San Jose, CA, USA). Factorial analysis was conducted for the fungicide treatments, and the means were compared using Tukey’s test at a 5% significance level (*p* < 0.05) with the software Assistat version 7.7 [30].

## 3. Results

### 3.1. Effect of Temperature on the Mycelial Growth Rate of Trichoderma *spp.*

**Figure 2:**
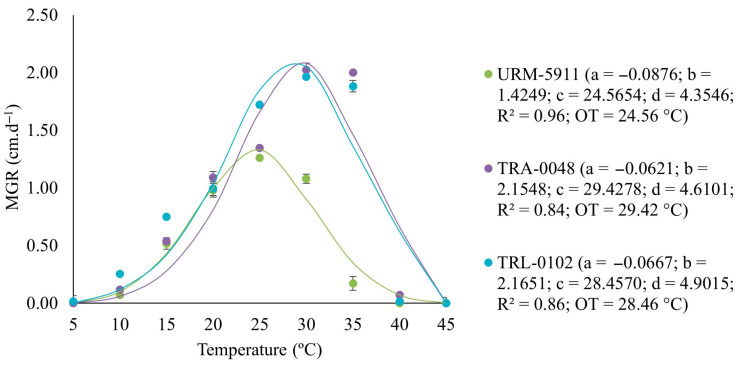
Regression equation, coefficient of determination (R^2^), and optimal temperature (OT) for mycelial growth of *Trichoderma* spp. isolates adjusted with values of the mycelial growth rate (MGR) at temperatures of 5, 10, 15, 20, 25, 30, 35, 40, and 45 °C. URM-5911 = *T. asperellum*; TRA-0048 = *T. asperellum*; TRL-0102 = *T. longibrachiatum*.

The selected cubic polynomial regression model used to describe mycelial growth at the different temperatures fitted the MGR data with an R^2^ ≥ 0.84 for all the evaluated *Trichoderma* spp. isolates (Figure 2).

**Figure 3:**
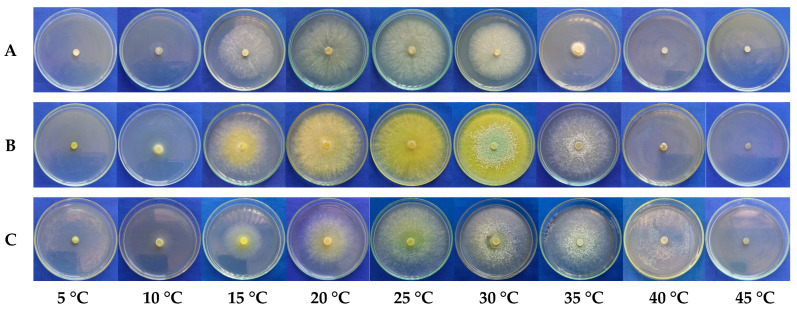
Mycelial growth of *Trichoderma* spp. isolates at temperatures of 5, 10, 15, 20, 25, 30, 35, 40, and 45 °C. (**A**) = URM-5911 (*T. asperellum*). (**B**) = TRA-0048 (*T. asperellum*). (**C**) = TRL-0102 (*T. longibrachiatum*).

The parameters of the fitted models (a, b, c, and d) revealed the differences in growth characteristics among the treatments. The three curves highlighted distinct optimal temperatures and growth ranges, with URM-5911 exhibiting a maximum growth at 24.56 °C, while those of TRA-0048 and TRL-0102 occurred at 29.42 and 28.46 °C, respectively. No mycelial growth was observed at 5 and 45 °C; however, the isolates showed growth when incubated at room temperature (±27 °C), indicating that they did not exert lethal effects on the isolates, although both the extreme temperatures inhibited mycelial development (Figure 3).

### 3.2. Effect of pH on the Mycelial Growth Rate of Trichoderma *spp.*

**Figure 4:**
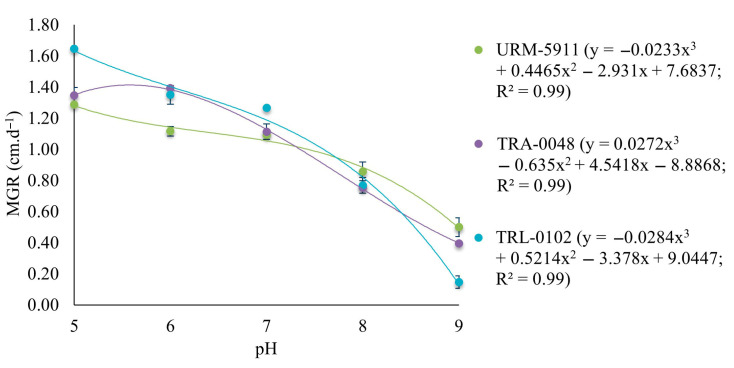
Regression equation and coefficient of determination (R^2^) for mycelial growth of *Trichoderma* spp. isolates. y = adjusted with values of mycelial growth rate (MGR) at pHs 5, 6, 7, 8, and 9. URM-5911 = *T. asperellum*; TRA-0048 = *T. asperellum*; TRL-0102 = *T. longibrachiatum*.

The cubic polynomial regression model selected to describe mycelial growth at the different pH levels fitted the MGR data with an R^2^ value of 0.99 for all the evaluated *Trichoderma* spp. isolates (Figure 4).

**Figure 5:**
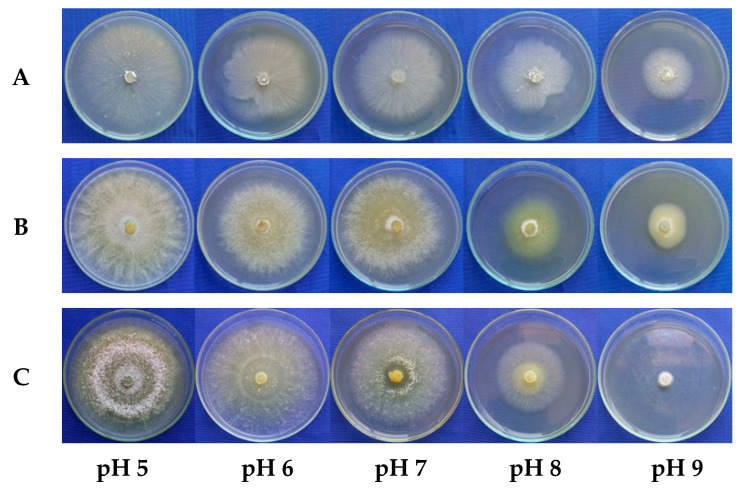
Mycelial growth of *Trichoderma* spp. isolates at pHs 5, 6, 7, 8, and 9. (**A**) = URM-5911 (*T. asperellum*). (**B**) = TRA-0048 (*T. asperellum*). (**C**) = TRL-0102 (*T. longibrachiatum*).

All the *Trichoderma* spp. isolates grew at all the tested pH levels. The isolates URM-5911 and TRL-0102 exhibited maximum mycelial growth at pH 5 (1.29 and 1.65 cm·d^−1^, respectively), while TRA-0048 showed maximum growth at pH 5.56 (1.40 cm·d^−1^). The regression curves also indicated that for all the treatments, mycelial growth gradually decreased as the pH deviated from the optimal range (from five to seven) (Figure 5).

### 3.3. Effect of Salinity on the Mycelial Growth Rate of Trichoderma *spp.*

**Figure 6:**
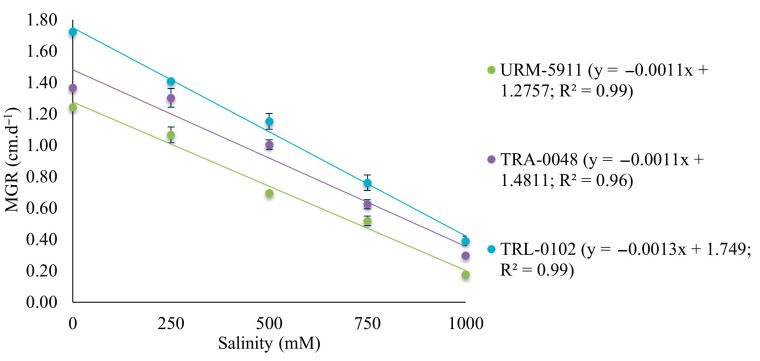
Regression equation and coefficient of determination (R^2^) for mycelial growth of *Trichoderma* spp. isolates. y = adjusted with values of mycelial growth rate (MGR) at NaCl concentrations of 0, 250, 500, 750, and 1000 mM. URM-5911 = *T. asperellum*; TRA-0048 = *T. asperellum*; TRL-0102 = *T. longibrachiatum*.

The linear regression model selected to describe mycelial growth at the different salinity concentrations fitted the MGR data with an R^2^ ≥ 0.96 for all the evaluated *Trichoderma* spp. isolates (Figure 6).

**Figure 7:**
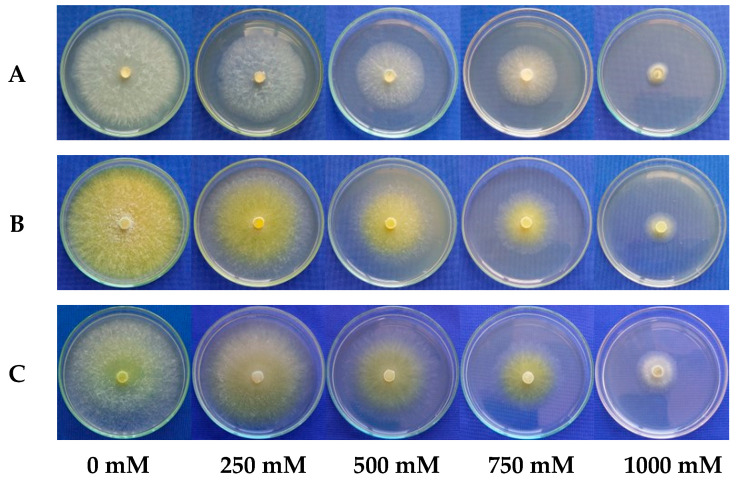
Mycelial growth of *Trichoderma* spp. isolates at NaCl concentrations of 0, 250, 500, 750, and 1000 mM. (**A**) = URM-5911 (*T. asperellum*). (**B**) = TRA-0048 (*T. asperellum*). (**C**) = TRL-0102 (*T. longibrachiatum*).

The mycelial growth rate decreased with increasing salinity, indicating a negative effect across all the evaluated *Trichoderma* spp. isolates. Mycelial growth for isolate URM-5911 was reduced by 85.5% at the highest tested concentration (1000 mM) compared to that of the control (0 mM). Isolates TRA-0048 and TRL-0102 showed reductions of 78.1 and 77.3%, respectively. Notably, TRL-0102 exhibited the highest initial growth rate (1.72 cm·d^−1^) and the greatest tolerance to salinity among the isolates, with a less pronounced decline in growth compared to those of the other treatments (Figure 7).

### 3.4. Sensitivity of Trichoderma *spp.* to Fungicides

**Figure 8:**
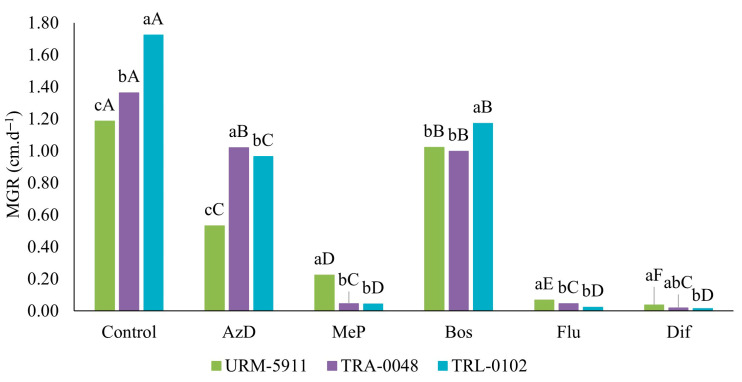
Effect of fungicides on mycelial growth rate (MGR) in cm·d^−1^ of *Trichoderma* spp. isolates. Lowercase letters compare isolates within same fungicide, and uppercase letters compare each isolate in different fungicide treatments by Tukey’s test at 5% probability level. AzD = Azoxystrobin + Difenoconazole; MeP = Metiram + Pyraclostrobin; Bos = Boscalid; Flu = Fludioxonil; Dif = Difenoconazole. URM-5911 = *T. asperellum*; TRA-0048 = *T. asperellum*; TRL-0102 = *T. longibrachiatum*.

A significant effect on the mycelial growth of *Trichoderma* spp. isolates was observed under the influence of the different fungicides (*p* < 0.05). However, this impact varied among the treatments (Figure 8).

**Figure 9:**
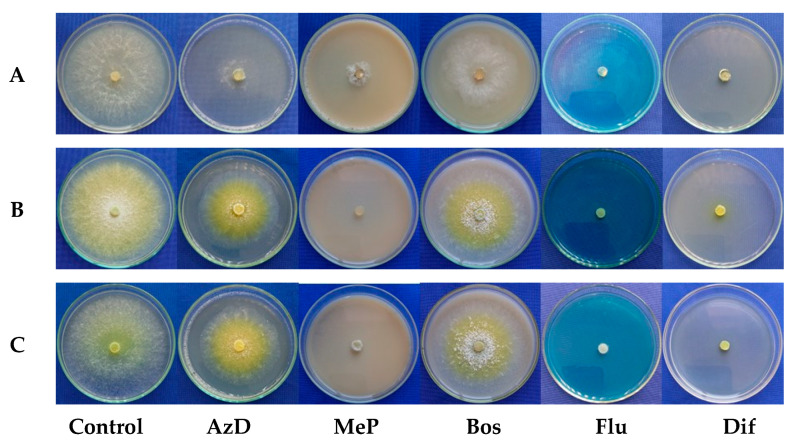
Mycelial growth of *Trichoderma* spp. isolates at fungicides Azoxystrobin + Difenoconazole (AzD), Metiram + Pyraclostrobin (MeP), Boscalid (Bos), Fludioxonil (Flu), and Difenoconazole (Dif). (**A**) = URM-5911 (*T. asperellum*). (**B**) = TRA-0048 (*T. asperellum*). (**C**) = TRL-0102 (*T. longibrachiatum*).

All the fungicides reduced the mycelial growth of the *Trichoderma* spp. isolates compared to that of the control. The treatments with AzD and Bos resulted in higher growth rates and less inhibition compared to those of the other tested fungicides. The URM-5911 isolate showed reductions of 55.5 and 14.3% for AzD and Bos, respectively, while TRA-0048 and TRL-0102 exhibited reductions of 25.0 and 44.2% (AzD) and 26.5 and 32.0% (Bos), respectively. Flu and Dif were the least selective fungicides, significantly suppressing the mycelial growth of all the *Trichoderma* spp. isolates, with growth rates below 0.07 cm·d^−1^. For MeP, URM-5911 showed a growth rate of 0.22 cm·d^−1^, while TRA-0048 and TRL-0102 reached only 0.05 and 0.04 cm·d^−1^, respectively. Mycelial growth was reduced for these fungicides by more than 80.0% compared to that of the control for all the tested isolates (Figure 9).

**Figure 10:**
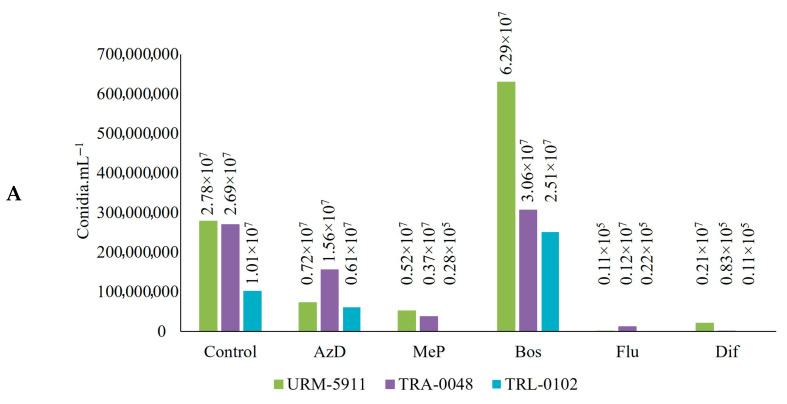

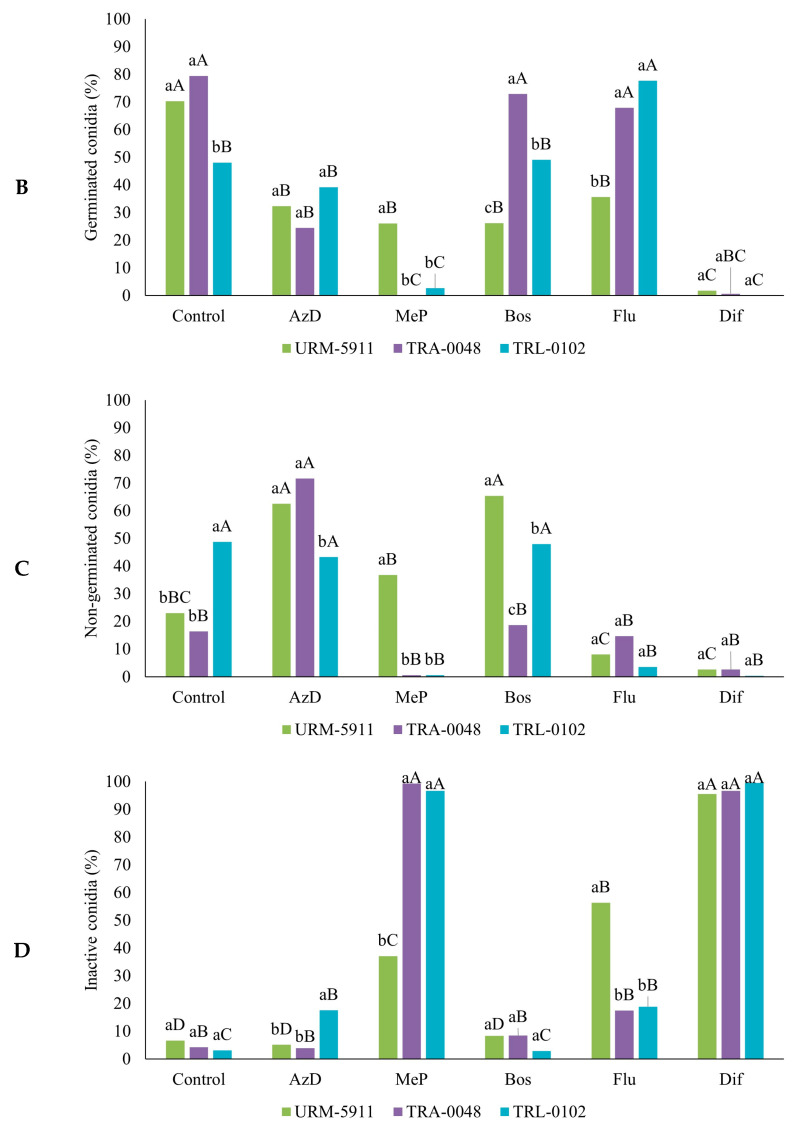
Effect of fungicides on production and viability of conidia of *Trichoderma* spp. isolates. (**A**) = Conidia production verified in 1 mL of conidium solution. (**B**) = Percentage of germinated active conidia after 20 h of incubation. (**C**) = Percentage of non-germinated active conidia after 20 h of incubation. (**D**) = Percentage of inactive conidia after 20 h of incubation. Lowercase letters compare isolates within same fungicide, and uppercase letters compare each isolate in different fungicide treatments by Tukey’s test at 5% probability level. AzD = Azoxystrobin + Difenoconazole; MeP = Metiram + Pyraclostrobin; Bos = Boscalid; Flu = Fludioxonil; Dif = Difenoconazole. URM-5911 = *T. asperellum*; TRA-0048 = *T. asperellum*; TRL-0102 = *T. longibrachiatum*.

A significant effect was also observed on the conidial production and viability of the *Trichoderma* spp. isolates under the influence of the different fungicides (*p* < 0.05), with variations among the treatments (Figure 10).

Except for Bos, all the other fungicides reduced the conidial production of the *Trichoderma* spp. isolates. Bos, however, resulted in a notable increase in the number of produced conidia, with isolate URM-5911 reaching 6.29 × 10^7^ conidia.mL^−1^, a 126.0% increase compared to that of the control treatment; TRA-0048 reaching 3.06 × 10^7^ conidia.mL^−1^, a 13.7% increase; and TRL-0102 reaching 2.51 × 10^7^ conidia.mL^−1^, a 148.5% increase (Figure 10A).

For isolate URM-5911, all the treatments containing fungicides reduced the percentage of germinated active conidia compared to that of the control, with the lowest percentage observed for the fungicide Flu (36.0%) (Figure 10B). The highest percentage of non-germinated conidia was observed in the treatments containing AzD (63.0%) and Bos (65.0%) (Figure 10C), while Dif resulted in the highest percentage of inactive conidia (96.0%) (Figure 10D).

For TRA-0048, the fungicides Bos (73.0%) and Flu (68.0%) resulted in percentages of germinated active conidia similar to those of the control (Figure 10B), while AzD was responsible for the highest percentage of non-germinated conidia (72.0%) (Figure 10C). MeP (99.0%) and Dif (97.0%) were responsible for the highest percentage of inactive conidia (Figure 10D).

For TRL-0102, the only fungicides that reduced the percentage of germinated active conidia compared to that of the control were MeP (3.0%) and Dif (0.0%) (Figure 10B), which also resulted in the highest percentage of inactive conidia (97.0 and 100.0%, respectively) (Figure 10D). The opposite effect was observed with Flu (78.0%), which provided a higher percentage of germinated active conidia than that observed in the control (Figure 10B). AzD (43.0%) and Bos (48.0%) were similar to the control in terms of the percentage of non-germinated conidia (Figure 10C).

## 4. Discussion

Several studies have investigated the impact of temperature on the mycelial growth of *Trichoderma* species, providing relevant information for interpreting the presented data. Oliveira et al. [2] observed that isolates of *T. asperellum* and *T. harzianum* exhibited optimal mycelial growth and reached the edge of the Petri dish within 72 h at 30 °C, a phenomenon also observed for the isolates evaluated in the present study. Other isolates, including the species *T. asperelloides*, showed maximum growth at 27 °C and biological control efficacy at temperatures ranging from 22 to 32 °C, suggesting that higher temperatures favor the activity of *Trichoderma* spp. against phytopathogens [31]. Parasitism was observed between 20 and 30 °C when evaluating the effect of temperatures from 15 to 30 °C on the ability of *T. harzianum* to parasitize *S. sclerotiorum* sclerotia, but was absent at 15 °C, indicating that temperatures below 20 °C may limit the effectiveness of these species as biological control agents [32].

These studies corroborate that temperature has a significant influence on the growth and effectiveness of *Trichoderma* spp. as biological control agents. Temperatures between 25 and 30 °C tend to be ideal for mycelial growth and antagonistic activity, while lower temperatures may reduce their efficacy. Although the absence of in vitro mycelial growth has been reported at 40 °C, under field conditions with adequate humidity, the fungus has shown full development at temperatures higher than those observed in laboratory settings [33,34]. In melon-cultivated soils, for example, temperature can range from 25 to 40 °C throughout the year, especially in semi-arid regions such as northeastern Brazil, which may affect the growth and activity of these *Trichoderma* species [35]. The optimal temperatures ranged from 24.56 to 29.42 °C for the tested commercial *Trichoderma*-based product isolates.

The pH significantly influenced mycelial growth, with the maximum growth rates generally occurring at values from around five to six. These results may vary according to the studied species and isolate. Optimal growth and conidia production for *T. harzianum* isolates, for example, were observed at pH 4 [18]. Zehra et al. [17] found that the growth and sporulation of different *Trichoderma* species were optimal at pH values between 4.1 and 8.6, with significant reductions outside this range. External pH can regulate growth, conidia production, colony morphology, and enzymatic activity related to mycoparasitism and protein secretion in these species [20]. Other studies have shown that the interaction of *Trichoderma* spp. with phytopathogens such as *S. sclerotiorum* and *F. solani* can be influenced by the medium pH, affecting its effectiveness as a biocontrol agent [21]. Several *Trichoderma* species, including *T. longibrachiatum*, demonstrated the ability to inhibit the mycelial growth of *R. solani* at pH 5.5, which was also associated with increased enzymatic activity and the production of volatile compounds capable of suppressing this pathogen [22]. These findings have significant practical implications. When using *Trichoderma* spp. as a biocontrol agent, the soil or substrate pH must be considered to maximize its effectiveness. In general, a pH range between five and seven is considered ideal, with specific variations depending on the species/isolate and the local environmental conditions.

Salinity negatively affected the mycelial growth of the treatments, with TRL-0102 demonstrating the highest tolerance among the evaluated isolates. These results are consistent with the current literature on the salt stress tolerance of *Trichoderma* species. Recent studies have identified isolates with high salinity tolerance. For instance, a *T*. *longibrachiatum* isolate (HL167) obtained from saline soils exhibited substantial growth in media containing up to 8% salt, indicating strong adaptation to such conditions [24]. Additionally, a *T*. *atroviride* isolate (HN082102.1) demonstrated the potential to alleviate salt stress and reduce *Fusarium*-induced root rot in cucumber [36]. *Trichoderma harzianum* was also reported to grow under very high NaCl concentrations (85–855 mM), with the marked stimulation of chlamydospore production at increasing salt levels, without impairing its saprophytic activity in soil [23].

Salt tolerance in *Trichoderma* is often associated with the ability to regulate osmotic balance and the expression of genes encoding ion transport systems, such as Na^+^/H^+^ antiporters and K^+^/Na^+^ transporters [37]. These mechanisms enable cells to maintain ionic homeostasis under high-salinity conditions, contributing to their survival and growth. The use of salt-tolerant *Trichoderma* spp. isolates has demonstrated agricultural benefits, particularly in salinity-affected areas. The inoculation of plants with *Trichoderma* species can enhance net photosynthesis, water use efficiency, and productivity under salt stress [38]. Furthermore, seed pre-treatment with *T. longibrachiatum* (TG1) increased the endogenous salicylic acid levels in wheat seedlings, promoting growth under saline conditions [39]. The results observed for the TRL-0102 isolate, which exhibited greater salinity tolerance, may be explained by mechanisms similar to those described in the literature, such as efficient osmotic regulation and adaptive ion transport systems. The identification and use of such isolates may represent a strategic approach for the development of effective biofertilizers in saline soils, thereby contributing to agricultural sustainability in regions affected by this type of abiotic stress.

The integration of biological agents associated with chemical fungicides is a common practice in IDM. However, compatibility between these agents is crucial to ensure the effectiveness of disease control and the sustainability of agricultural systems. The fungicides based on Azoxystrobin + Difenoconazole and Boscalid, which showed the best results in the present analysis, contain active ingredients that have previously been reported to be compatible with *Trichoderma* spp. [28,40]. Other active ingredients, such as Fluazinam, Fludioxonil, Mancozeb, Copper oxychloride, and Thiram, among others, have also been reported as compatible and may be incorporated into IDM strategies for the control of soilborne phytopathogens [26,27,40]. This compatibility may vary depending on the interaction between the active ingredient and the formulation of the *Trichoderma*-based products used. Branco et al. [25], for example, reported incompatibility between fungicides containing different active ingredients, including Fluazinam and Fludioxonil, and a *T*. *harzianum* isolate obtained from the commercial product Ecotrip WP^®^. Khirallah et al. [1] observed the low-to-moderate compatibility of Boscalid-based fungicides regarding conidial production and germination in different *Trichoderma* species.

The appropriate selection of compatible products can maximize disease control efficacy and minimize negative impacts on BCAs. For the isolates obtained from the evaluated *Trichoderma*-based products, the fungicides Azoxystrobin + Difenoconazole and Boscalid may be compatible for use in integrated disease management programs. These findings highlight the importance of conducting compatibility tests before combining chemical fungicides with *Trichoderma* species.

## 5. Conclusions

The evaluated *Trichoderma* spp. isolates demonstrated adaptability to environmental and chemical variations, including broad tolerance to different temperatures, pH levels, and salinity conditions, as well as differentiated responses to synthetic fungicides. These characteristics highlight their potential application as biological control agents in diverse agricultural contexts, especially in semi-arid regions such as northeastern Brazil.

The identification of isolates with enhanced stress tolerance provides a strategic basis for the development of more effective and resilient biopreparations. The compatibility of certain fungicides, particularly Boscalid and Azoxystrobin + Difenoconazole, supports their incorporation into integrated disease management programs, promoting more sustainable and environmentally friendly crop protection strategies.

These findings contribute to expanding the use of *Trichoderma*-based bioproducts by providing selection criteria for robust and efficient isolates suited to field application. They also strengthen the foundation for the formulation of stable and compatible commercial products tailored to local edaphoclimatic conditions. Field validation remains essential to confirm the in vitro results and to evaluate the interactions with native soil microbiota and pathogen populations.

## Figures and Tables

**Table 1:** *Trichoderma*-based product isolates used in this study.

Isolate Code	*Trichoderma* Species	Product	Formulation ^1^	Company
URM-5911	*T. asperellum*	Quality^®^ WG	WG	Lallemand Soluções Biológicas Ltd., Piracicaba, Brazil
TRA-0048	*T. asperellum*	TrichobiolMax	CS	Biofungi Controle Biológico, Eunápolis, Brazil
TRL-0102	*T. longibrachiatum*	TrichonemateMax	CS	Biofungi Controle Biológico, Eunápolis, Brazil

^1^ WG = water dispersible granule; CS = concentrated suspension.

## Data Availability

The original contributions presented in the study are included in the article, further inquiries can be directed to the corresponding authors.

## Abbreviations

The following abbreviations are used in this manuscript:

AzD	Azoxystrobin + Difenoconazole
BCAs	Biological control agents
BOD	Biochemical oxygen demand
Bos	Boscalid
CS	Concentrated suspension
Dif	Difenoconazole
EC	Emulsifiable concentrate
Flu	Fludioxonil
IDM	Integrated disease management
MeP	Metiram + Pyraclostrobin
MGR	Mycelial growth rate
NaCl	Sodium chloride
NaOH	Sodium hydroxide
OT	Optimal temperature
PDA	Potato dextrose agar
WG	Water dispersible granule
